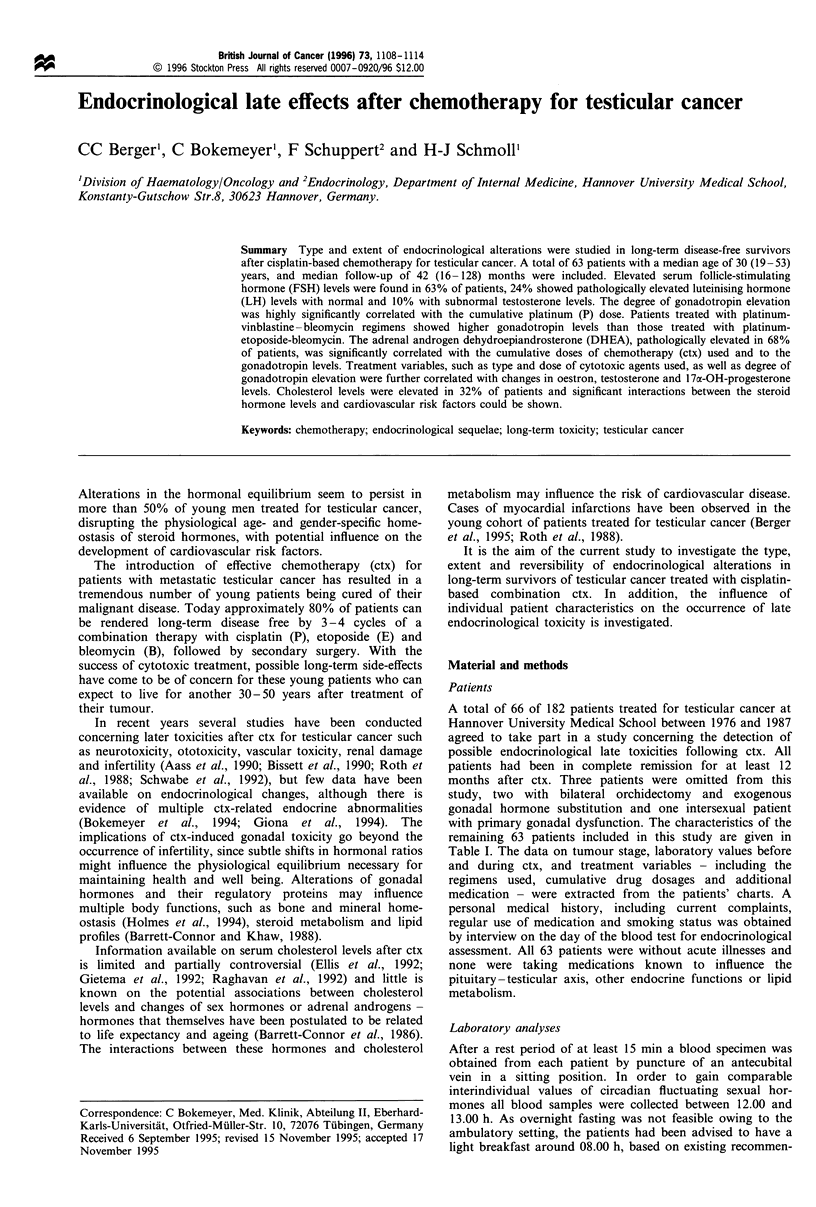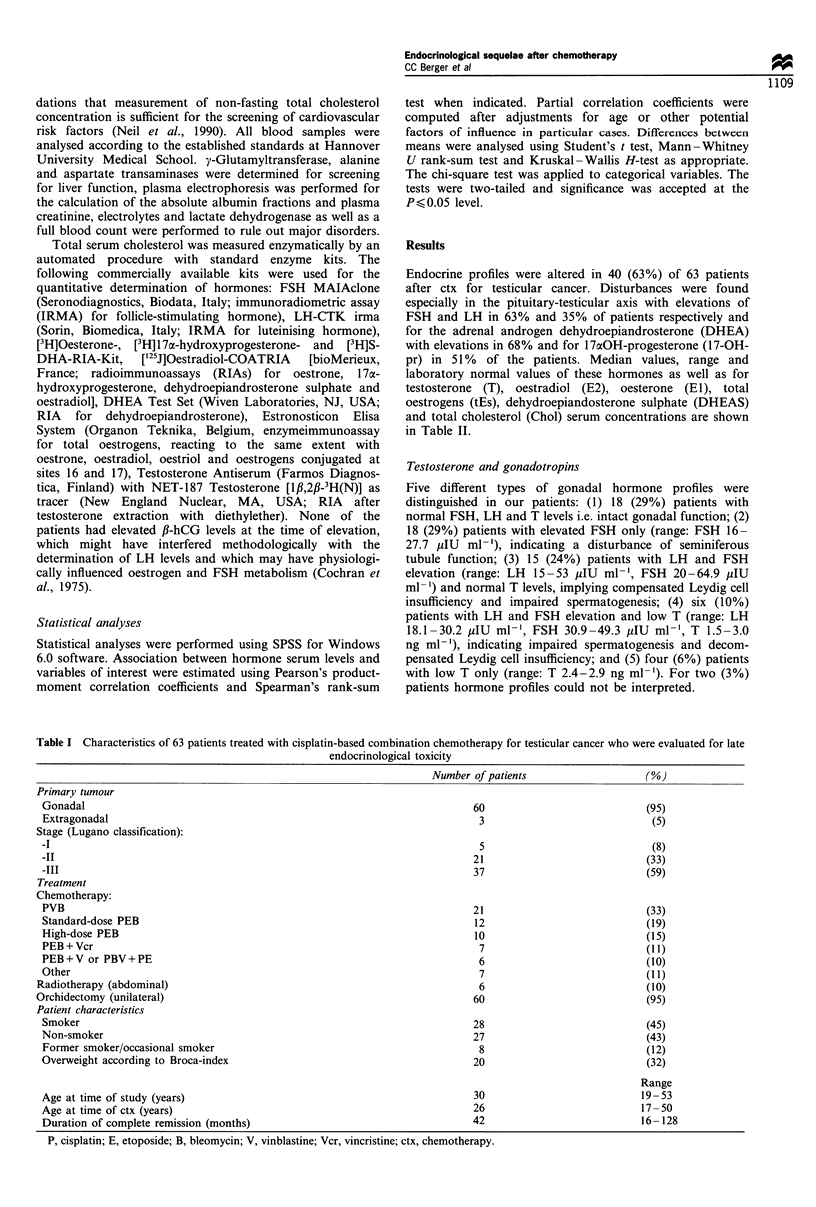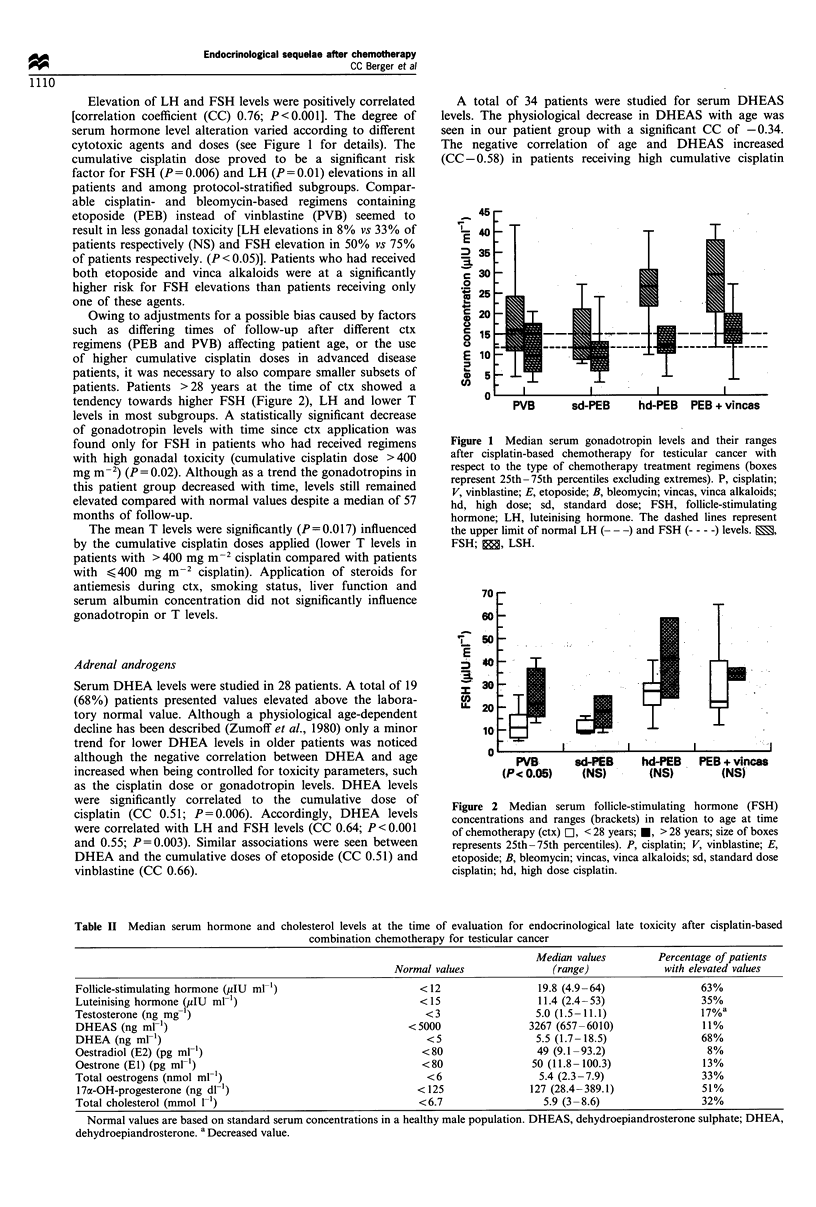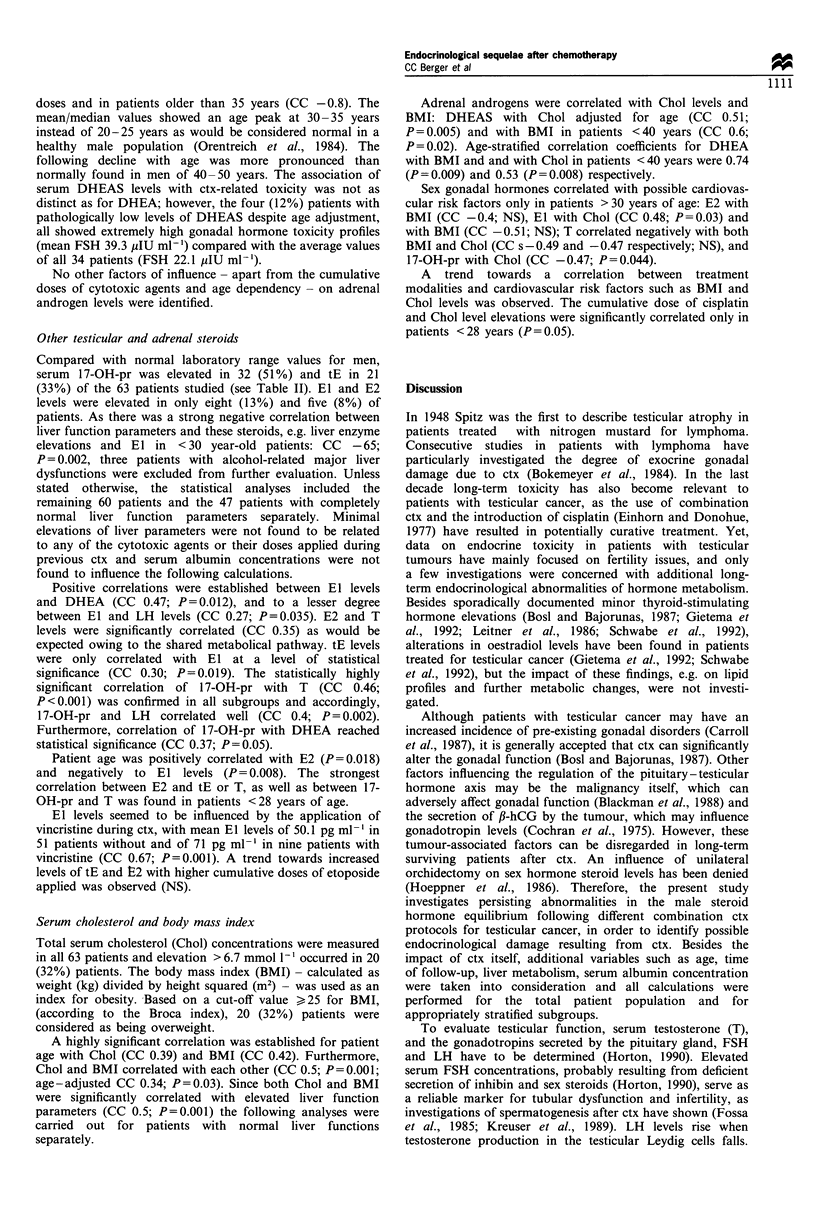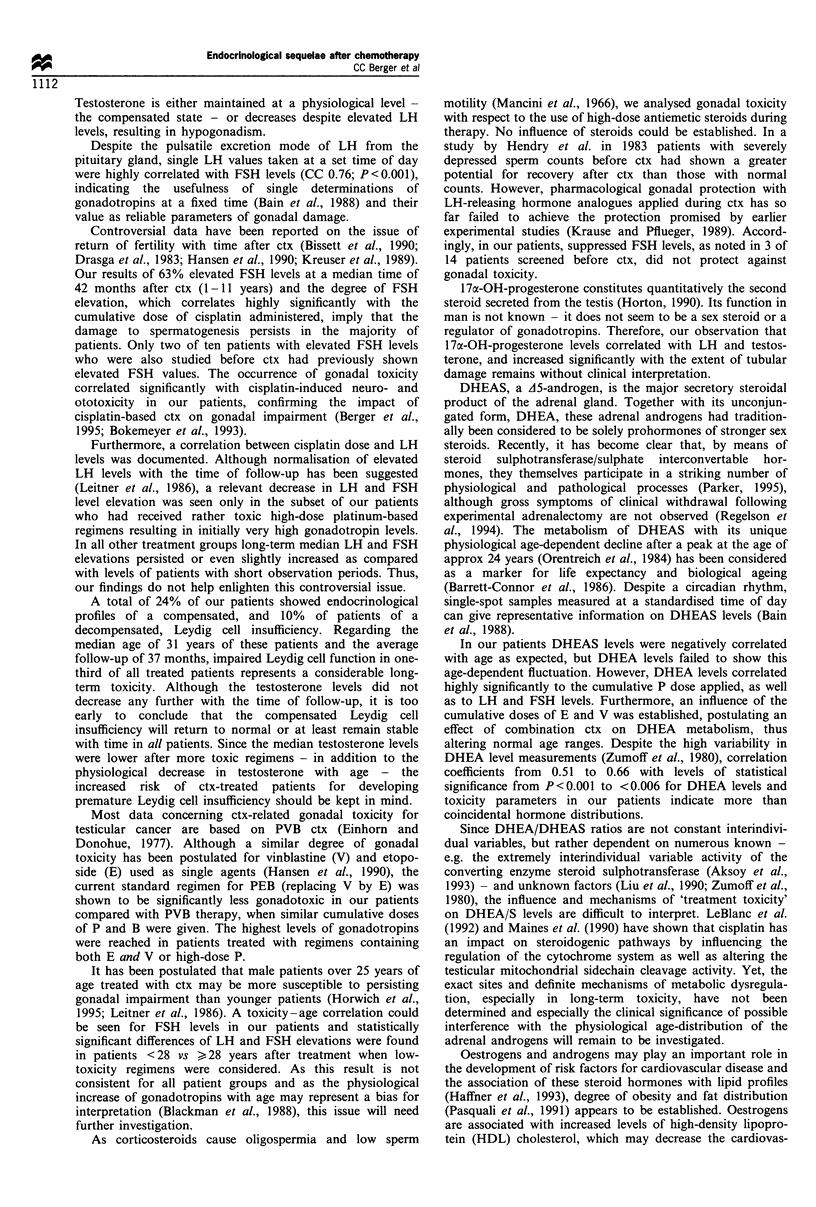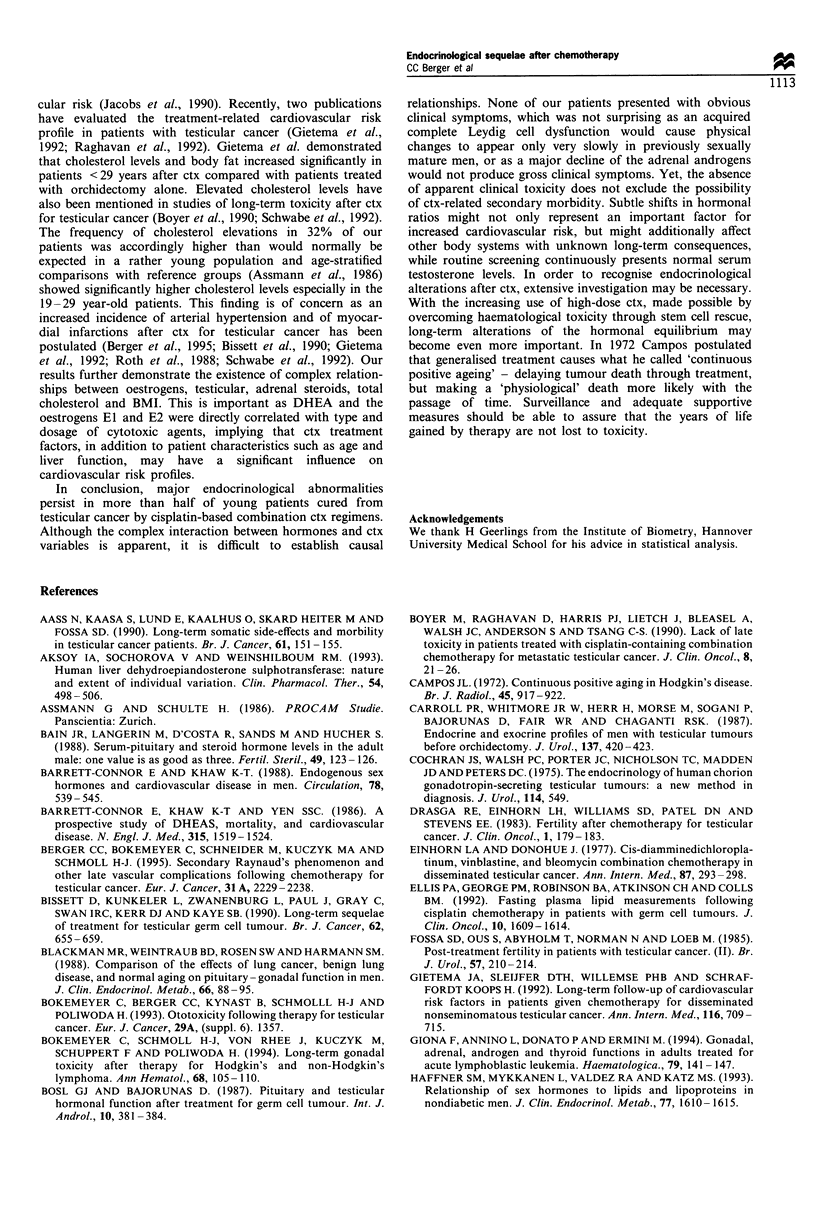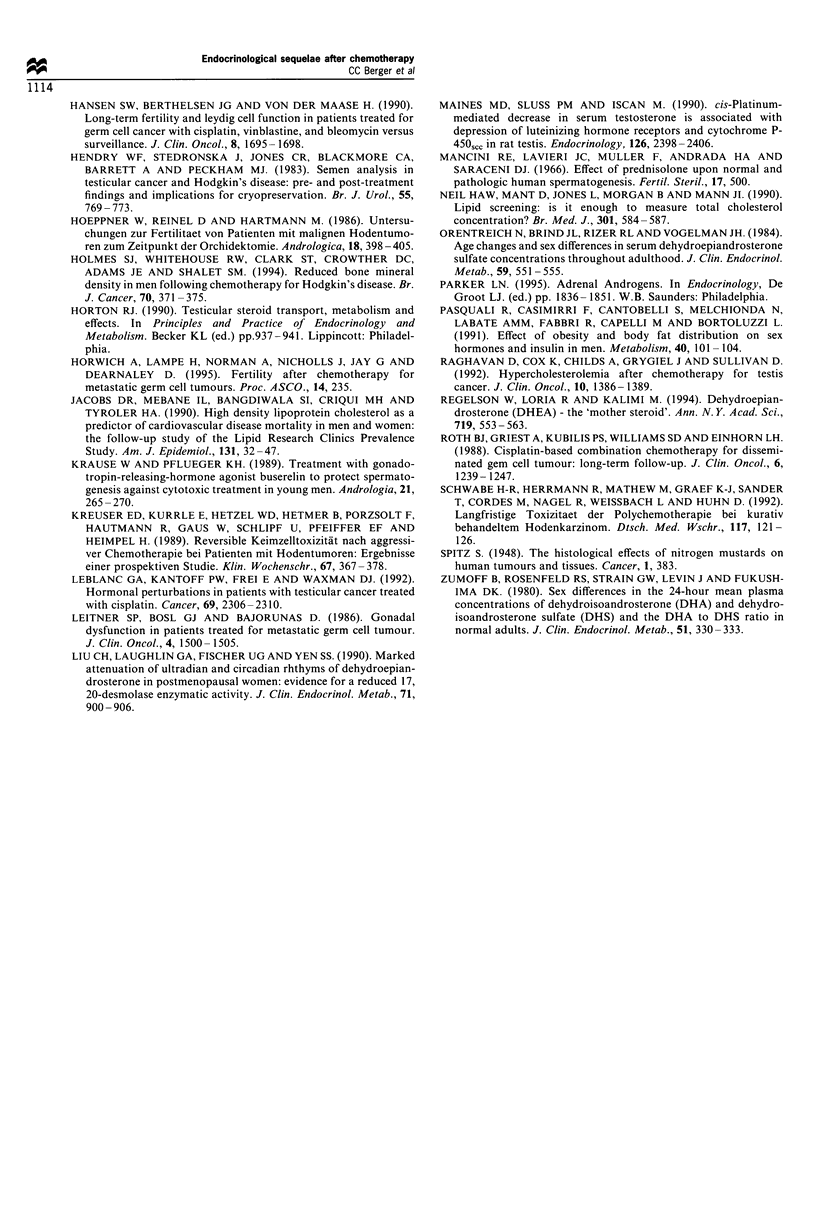# Endocrinological late effects after chemotherapy for testicular cancer.

**DOI:** 10.1038/bjc.1996.213

**Published:** 1996-05

**Authors:** C. C. Berger, C. Bokemeyer, F. Schuppert, H. J. Schmoll

**Affiliations:** Division of Haematology/Oncology, Hannover University Medical School, Germany.

## Abstract

Type and extent of endocrinological alterations were studied in long-term disease-free survivors after cisplatin-based chemotherapy for testicular cancer. A total of 63 patients with a median age of 30 (19-53) years, and median follow-up of 42 (16-128) months were included. Elevated serum follicle-stimulating hormone (FSH) levels were found in 63% of patients, 24% showed pathologically elevated luteinising hormone (LH) levels with normal and 10% with subnormal testosterone levels. The degree of gonadotropin elevation was highly significantly correlated with the cumulative platinum (P) dose. Patients treated with platinum-vinblastine-bleomycin regimens showed higher gonadotropin levels than those treated with platinum-etoposide-bleomycin. The adrenal androgen dehydroepiandrosterone (DHEA), pathologically elevated in 68% of patients, was significantly correlated with the cumulative doses of chemotherapy (ctx) used and to the gonadotropin levels. Treatment variables, such as type and dose of cytotoxic agents used, as well as degree of gonadotropin elevation were further correlated with changes in oestron, testosterone and 17 alpha-OH-progesterone levels. Cholesterol levels were elevated in 32% of patients and significant interactions between the steroid hormone levels and cardiovascular risk factors could be shown.


British Journal of Cancer (1996) 73, 1108-1114
© 1996 Stockton Press All rights reserved 0007-0920/96 $12.00

### Endocrinological late effects after chemotherapy for testicular cancer

CC Berger[1], C Bokemeyer[1], F Schuppert[2] and H-J Schmoll[1]

[1]*Division of Haematology/Oncology and* [2]*Endocrinology, Department of Internal Medicine, Hannover University Medical School, Konstanty-Gutschow Str.8, 30623 Hannover, Germany.*

**Summary** Type and extent of endocrinological alterations were studied in long-term disease-free survivors after cisplatin-based chemotherapy for testicular cancer. A total of 63 patients with a median age of 30 (19–53) years, and median follow-up of 42 (16–128) months were included. Elevated serum follicle-stimulating hormone (FSH) levels were found in 63% of patients, 24% showed pathologically elevated luteinising hormone (LH) levels with normal and 10% with subnormal testosterone levels. The degree of gonadotropin elevation was highly significantly correlated with the cumulative platinum (P) dose. Patients treated with platinum-vinblastine–bleomycin regimens showed higher gonadotropin levels than those treated with platinum-etoposide-bleomycin. The adrenal androgen dehydroepiandrosterone (DHEA), pathologically elevated in 68% of patients, was significantly correlated with the cumulative doses of chemotherapy (ctx) used and to the gonadotropin levels. Treatment variables, such as type and dose of cytotoxic agents used, as well as degree of gonadotropin elevation were further correlated with changes in oestron, testosterone and 17α-OH-progesterone levels. Cholesterol levels were elevated in 32% of patients and significant interactions between the steroid hormone levels and cardiovascular risk factors could be shown.

**Keywords:** chemotherapy; endocrinological sequelae; long-term toxicity; testicular cancer

Alterations in the hormonal equilibrium seem to persist in more than 50% of young men treated for testicular cancer, disrupting the physiological age- and gender-specific homeostasis of steroid hormones, with potential influence on the development of cardiovascular risk factors.

The introduction of effective chemotherapy (ctx) for patients with metastatic testicular cancer has resulted in a tremendous number of young patients being cured of their malignant disease. Today approximately 80% of patients can be rendered long-term disease free by 3–4 cycles of a combination therapy with cisplatin (P), etoposide (E) and bleomycin (B), followed by secondary surgery. With the success of cytotoxic treatment, possible long-term side-effects have come to be of concern for these young patients who can expect to live for another 30–50 years after treatment of their tumour.

In recent years several studies have been conducted concerning later toxicities after ctx for testicular cancer such as neurotoxicity, ototoxicity, vascular toxicity, renal damage and infertility (Aass *et al.*, 1990; Bissett *et al.*, 1990; Roth *et al.*, 1988; Schwabe *et al.*, 1992), but few data have been available on endocrinological changes, although there is evidence of multiple ctx-related endocrine abnormalities (Bokemeyer *et al.*, 1994; Giona *et al.*, 1994). The implications of ctx-induced gonadal toxicity go beyond the occurrence of infertility, since subtle shifts in hormonal ratios might influence the physiological equilibrium necessary for maintaining health and well being. Alterations of gonadal hormones and their regulatory proteins may influence multiple body functions, such as bone and mineral homeostasis (Holmes *et al.*, 1994), steroid metabolism and lipid profiles (Barrett-Connor and Khaw, 1988).

Information available on serum cholesterol levels after ctx is limited and partially controversial (Ellis *et al.*, 1992; Gietema *et al.*, 1992; Raghavan *et al.*, 1992) and little is known on the potential associations between cholesterol levels and changes of sex hormones or adrenal androgens – hormones that themselves have been postulated to be related to life expectancy and ageing (Barrett-Connor *et al.*, 1986). The interactions between these hormones and cholesterol metabolism may influence the risk of cardiovascular disease. Cases of myocardial infarctions have been observed in the young cohort of patients treated for testicular cancer (Berger *et al.*, 1995; Roth *et al.*, 1988).

It is the aim of the current study to investigate the type, extent and reversibility of endocrinological alterations in long-term survivors of testicular cancer treated with cisplatin-based combination ctx. In addition, the influence of individual patient characteristics on the occurrence of late endocrinological toxicity is investigated.

### Material and methods

### *Patients*

A total of 66 of 182 patients treated for testicular cancer at Hannover University Medical School between 1976 and 1987 agreed to take part in a study concerning the detection of possible endocrinological late toxicities following ctx. All patients had been in complete remission for at least 12 months after ctx. Three patients were omitted from this study, two with bilateral orchidectomy and exogenous gonadal hormone substitution and one intersexual patient with primary gonadal dysfunction. The characteristics of the remaining 63 patients included in this study are given in Table I. The data on tumour stage, laboratory values before and during ctx, and treatment variables – including the regimens used, cumulative drug dosages and additional medication – were extracted from the patients' charts. A personal medical history, including current complaints, regular use of medication and smoking status was obtained by interview on the day of the blood test for endocrinological assessment. All 63 patients were without acute illnesses and none were taking medications known to influence the pituitary–testicular axis, other endocrine functions or lipid metabolism.

### *Laboratory analyses*

After a rest period of at least 15 min a blood specimen was obtained from each patient by puncture of an antecubital vein in a sitting position. In order to gain comparable interindividual values of circadian fluctuating sexual hormones all blood samples were collected between 12.00 and 13.00 h. As overnight fasting was not feasible owing to the ambulatory setting, the patients had been advised to have a light breakfast around 08.00 h, based on existing recommen-

Correspondence: C Bokemeyer, Med. Klinik, Abteilung II, Eberhard-Karls-Universität, Otfried-Müller-Str. 10, 72076 Tübingen, Germany
Received 6 September 1995; revised 15 November 1995; accepted 17 November 1995

dations that measurement of non-fasting total cholesterol concentration is sufficient for the screening of cardiovascular risk factors (Neil *et al.*, 1990). All blood samples were analysed according to the established standards at Hannover University Medical School. $\gamma$-Glutamyltransferase, alanine and aspartate transaminases were determined for screening for liver function, plasma electrophoresis was performed for the calculation of the absolute albumin fractions and plasma creatinine, electrolytes and lactate dehydrogenase as well as a full blood count were performed to rule out major disorders.

Total serum cholesterol was measured enzymatically by an automated procedure with standard enzyme kits. The following commercially available kits were used for the quantitative determination of hormones: FSH MAIAclone (Seronodiagnostics, Biodata, Italy; immunoradiometric assay (IRMA) for follicle-stimulating hormone), LH-CTK irma (Sorin, Biomedica, Italy; IRMA for luteinising hormone), [$^3$H]Oesterone-, [$^3$H]17$\alpha$-hydroxyprogesterone- and [$^3$H]S-DHA-RIA-Kit, [$^{125}$J]Oestradiol-COATRIA [bioMerieux, France; radioimmunoassays (RIAs) for oestrone, 17$\alpha$-hydroxyprogesterone, dehydroepiandrosterone sulphate and oestradiol], DHEA Test Set (Wiven Laboratories, NJ, USA; RIA for dehydroepiandrosterone), Estronosticon Elisa System (Organon Teknika, Belgium, enzymeimmunoassay for total oestrogens, reacting to the same extent with oestrone, oestradiol, oestriol and oestrogens conjugated at sites 16 and 17), Testosterone Antiserum (Farmos Diagnostica, Finland) with NET-187 Testosterone [1$\beta$,2$\beta$-$^3$H(N)] as tracer (New England Nuclear, MA, USA; RIA after testosterone extraction with diethylether). None of the patients had elevated $\beta$-hCG levels at the time of elevation, which might have interfered methodologically with the determination of LH levels and which may have physiologically influenced oestrogen and FSH metabolism (Cochran *et al.*, 1975).

### *Statistical analyses*

Statistical analyses were performed using SPSS for Windows 6.0 software. Association between hormone serum levels and variables of interest were estimated using Pearson's product-moment correlation coefficients and Spearman's rank-sum test when indicated. Partial correlation coefficients were computed after adjustments for age or other potential factors of influence in particular cases. Differences between means were analysed using Student's $t$ test, Mann–Whitney $U$ rank-sum test and Kruskal–Wallis $H$-test as appropriate. The chi-square test was applied to categorical variables. The tests were two-tailed and significance was accepted at the $P \leqslant 0.05$ level.

### Results

Endocrine profiles were altered in 40 (63%) of 63 patients after ctx for testicular cancer. Disturbances were found especially in the pituitary-testicular axis with elevations of FSH and LH in 63% and 35% of patients respectively and for the adrenal androgen dehydroepiandrosterone (DHEA) with elevations in 68% and for 17$\alpha$OH-progesterone (17-OH-pr) in 51% of the patients. Median values, range and laboratory normal values of these hormones as well as for testosterone (T), oestradiol (E2), oesterone (E1), total oestrogens (tEs), dehydroepiandosterone sulphate (DHEAS) and total cholesterol (Chol) serum concentrations are shown in Table II.

### *Testosterone and gonadotropins*

Five different types of gonadal hormone profiles were distinguished in our patients: (1) 18 (29%) patients with normal FSH, LH and T levels i.e. intact gonadal function; (2) 18 (29%) patients with elevated FSH only (range: FSH 16–27.7 $\mu$IU ml$^{-1}$), indicating a disturbance of seminiferous tubule function; (3) 15 (24%) patients with LH and FSH elevation (range: LH 15–53 $\mu$IU ml$^{-1}$, FSH 20–64.9 $\mu$IU ml$^{-1}$) and normal T levels, implying compensated Leydig cell insufficiency and impaired spermatogenesis; (4) six (10%) patients with LH and FSH elevation and low T (range: LH 18.1–30.2 $\mu$IU ml$^{-1}$, FSH 30.9–49.3 $\mu$IU ml$^{-1}$, T 1.5–3.0 ng ml$^{-1}$), indicating impaired spermatogenesis and decompensated Leydig cell insufficiency; and (5) four (6%) patients with low T only (range: T 2.4–2.9 ng ml$^{-1}$). For two (3%) patients hormone profiles could not be interpreted.

**Table I** Characteristics of 63 patients treated with cisplatin-based combination chemotherapy for testicular cancer who were evaluated for late endocrinological toxicity

| | *Number of patients* | *(%)* |
|---|---|---|
| *Primary tumour* | | |
| Gonadal | 60 | (95) |
| Extragonadal | 3 | (5) |
| Stage (Lugano classification): | | |
| -I | 5 | (8) |
| -II | 21 | (33) |
| -III | 37 | (59) |
| *Treatment* | | |
| Chemotherapy: | | |
| PVB | 21 | (33) |
| Standard-dose PEB | 12 | (19) |
| High-dose PEB | 10 | (15) |
| PEB + Vcr | 7 | (11) |
| PEB + V or PBV + PE | 6 | (10) |
| Other | 7 | (11) |
| Radiotherapy (abdominal) | 6 | (10) |
| Orchidectomy (unilateral) | 60 | (95) |
| *Patient characteristics* | | |
| Smoker | 28 | (45) |
| Non-smoker | 27 | (43) |
| Former smoker/occasional smoker | 8 | (12) |
| Overweight according to Broca-index | 20 | (32) |
| | | Range |
| Age at time of study (years) | 30 | 19–53 |
| Age at time of ctx (years) | 26 | 17–50 |
| Duration of complete remission (months) | 42 | 16–128 |

P, cisplatin; E, etoposide; B, bleomycin; V, vinblastine; Vcr, vincristine; ctx, chemotherapy.

Elevation of LH and FSH levels were positively correlated [correlation coefficient (CC) 0.76; $P<0.001$]. The degree of serum hormone level alteration varied according to different cytotoxic agents and doses (see Figure 1 for details). The cumulative cisplatin dose proved to be a significant risk factor for FSH ($P=0.006$) and LH ($P=0.01$) elevations in all patients and among protocol-stratified subgroups. Comparable cisplatin- and bleomycin-based regimens containing etoposide (PEB) instead of vinblastine (PVB) seemed to result in less gonadal toxicity [LH elevations in 8% *vs* 33% of patients respectively (NS) and FSH elevation in 50% *vs* 75% of patients respectively. ($P<0.05$)]. Patients who had received both etoposide and vinca alkaloids were at a significantly higher risk for FSH elevations than patients receiving only one of these agents.

Owing to adjustments for a possible bias caused by factors such as differing times of follow-up after different ctx regimens (PEB and PVB) affecting patient age, or the use of higher cumulative cisplatin doses in advanced disease patients, it was necessary to also compare smaller subsets of patients. Patients >28 years at the time of ctx showed a tendency towards higher FSH (Figure 2), LH and lower T levels in most subgroups. A statistically significant decrease of gonadotropin levels with time since ctx application was found only for FSH in patients who had received regimens with high gonadal toxicity (cumulative cisplatin dose >400 mg $m^{-2}$) ($P=0.02$). Although as a trend the gonadotropins in this patient group decreased with time, levels still remained elevated compared with normal values despite a median of 57 months of follow-up.

The mean T levels were significantly ($P=0.017$) influenced by the cumulative cisplatin doses applied (lower T levels in patients with >400 mg $m^{-2}$ cisplatin compared with patients with $\leqslant$400 mg $m^{-2}$ cisplatin). Application of steroids for antiemesis during ctx, smoking status, liver function and serum albumin concentration did not significantly influence gonadotropin or T levels.

### *Adrenal androgens*

Serum DHEA levels were studied in 28 patients. A total of 19 (68%) patients presented values elevated above the laboratory normal value. Although a physiological age-dependent decline has been described (Zumoff *et al.*, 1980) only a minor trend for lower DHEA levels in older patients was noticed although the negative correlation between DHEA and age increased when being controlled for toxicity parameters, such as the cisplatin dose or gonadotropin levels. DHEA levels were significantly correlated to the cumulative dose of cisplatin (CC 0.51; $P=0.006$). Accordingly, DHEA levels were correlated with LH and FSH levels (CC 0.64; $P<0.001$ and 0.55; $P=0.003$). Similar associations were seen between DHEA and the cumulative doses of etoposide (CC 0.51) and vinblastine (CC 0.66).

A total of 34 patients were studied for serum DHEAS levels. The physiological decrease in DHEAS with age was seen in our patient group with a significant CC of $-0.34$. The negative correlation of age and DHEAS increased (CC$-0.58$) in patients receiving high cumulative cisplatin

**Figure 1** Median serum gonadotropin levels and their ranges after cisplatin-based chemotherapy for testicular cancer with respect to the type of chemotherapy treatment regimens (boxes represent 25th–75th percentiles excluding extremes). P, cisplatin; *V*, vinblastine; *E*, etoposide; *B*, bleomycin; vincas, vinca alkaloids; hd, high dose; sd, standard dose; FSH, follicle-stimulating hormone; LH, luteinising hormone. The dashed lines represent the upper limit of normal LH (– – –) and FSH (- - - -) levels. ▧, FSH; ▩, LSH.

**Figure 2** Median serum follicle-stimulating hormone (FSH) concentrations and ranges (brackets) in relation to age at time of chemotherapy (ctx) □, <28 years; ■, >28 years; size of boxes represents 25th–75th percentiles). *P*, cisplatin; *V*, vinblastine; *E*, etoposide; *B*, bleomycin; vincas, vinca alkaloids; sd, standard dose cisplatin; hd, high dose cisplatin.

**Table II** Median serum hormone and cholesterol levels at the time of evaluation for endocrinological late toxicity after cisplatin-based combination chemotherapy for testicular cancer

| | *Normal values* | *Median values (range)* | *Percentage of patients with elevated values* |
|---|---|---|---|
| Follicle-stimulating hormone (µIU $ml^{-1}$) | <12 | 19.8 (4.9–64) | 63% |
| Luteinising hormone (µIU $ml^{-1}$) | <15 | 11.4 (2.4–53) | 35% |
| Testosterone (ng $mg^{-1}$) | <3 | 5.0 (1.5–11.1) | 17%[a] |
| DHEAS (ng $ml^{-1}$) | <5000 | 3267 (657–6010) | 11% |
| DHEA (ng $ml^{-1}$) | <5 | 5.5 (1.7–18.5) | 68% |
| Oestradiol (E2) (pg $ml^{-1}$) | <80 | 49 (9.1–93.2) | 8% |
| Oestrone (E1) (pg $ml^{-1}$) | <80 | 50 (11.8–100.3) | 13% |
| Total oestrogens (nmol $ml^{-1}$) | <6 | 5.4 (2.3–7.9) | 33% |
| 17α-OH-progesterone (ng $dl^{-1}$) | <125 | 127 (28.4–389.1) | 51% |
| Total cholesterol (mmol $l^{-1}$) | <6.7 | 5.9 (3–8.6) | 32% |

Normal values are based on standard serum concentrations in a healthy male population. DHEAS, dehydroepiandrosterone sulphate; DHEA, dehydroepiandrosterone. [a] Decreased value.

doses and in patients older than 35 years (CC −0.8). The mean/median values showed an age peak at 30–35 years instead of 20–25 years as would be considered normal in a healthy male population (Orentreich *et al.*, 1984). The following decline with age was more pronounced than normally found in men of 40–50 years. The association of serum DHEAS levels with ctx-related toxicity was not as distinct as for DHEA; however, the four (12%) patients with pathologically low levels of DHEAS despite age adjustment, all showed extremely high gonadal hormone toxicity profiles (mean FSH 39.3 $\mu$IU $ml^{-1}$) compared with the average values of all 34 patients (FSH 22.1 $\mu$IU $ml^{-1}$).

No other factors of influence – apart from the cumulative doses of cytotoxic agents and age dependency – on adrenal androgen levels were identified.

### *Other testicular and adrenal steroids*

Compared with normal laboratory range values for men, serum 17-OH-pr was elevated in 32 (51%) and tE in 21 (33%) of the 63 patients studied (see Table II). E1 and E2 levels were elevated in only eight (13%) and five (8%) of patients. As there was a strong negative correlation between liver function parameters and these steroids, e.g. liver enzyme elevations and E1 in <30 year-old patients: CC −65; $P=0.002$, three patients with alcohol-related major liver dysfunctions were excluded from further evaluation. Unless stated otherwise, the statistical analyses included the remaining 60 patients and the 47 patients with completely normal liver function parameters separately. Minimal elevations of liver parameters were not found to be related to any of the cytotoxic agents or their doses applied during previous ctx and serum albumin concentrations were not found to influence the following calculations.

Positive correlations were established between E1 levels and DHEA (CC 0.47; $P=0.012$), and to a lesser degree between E1 and LH levels (CC 0.27; $P=0.035$). E2 and T levels were significantly correlated (CC 0.35) as would be expected owing to the shared metabolical pathway. tE levels were only correlated with E1 at a level of statistical significance (CC 0.30; $P=0.019$). The statistically highly significant correlation of 17-OH-pr with T (CC 0.46; $P<0.001$) was confirmed in all subgroups and accordingly, 17-OH-pr and LH correlated well (CC 0.4; $P=0.002$). Furthermore, correlation of 17-OH-pr with DHEA reached statistical significance (CC 0.37; $P=0.05$).

Patient age was positively correlated with E2 ($P=0.018$) and negatively to E1 levels ($P=0.008$). The strongest correlation between E2 and tE or T, as well as between 17-OH-pr and T was found in patients <28 years of age.

E1 levels seemed to be influenced by the application of vincristine during ctx, with mean E1 levels of 50.1 pg $ml^{-1}$ in 51 patients without and of 71 pg $ml^{-1}$ in nine patients with vincristine (CC 0.67; $P=0.001$). A trend towards increased levels of tE and E2 with higher cumulative doses of etoposide applied was observed (NS).

### *Serum cholesterol and body mass index*

Total serum cholesterol (Chol) concentrations were measured in all 63 patients and elevation >6.7 mmol $l^{-1}$ occurred in 20 (32%) patients. The body mass index (BMI) – calculated as weight (kg) divided by height squared ($m^2$) – was used as an index for obesity. Based on a cut-off value ⩾25 for BMI, (according to the Broca index), 20 (32%) patients were considered as being overweight.

A highly significant correlation was established for patient age with Chol (CC 0.39) and BMI (CC 0.42). Furthermore, Chol and BMI correlated with each other (CC 0.5; $P=0.001$; age-adjusted CC 0.34; $P=0.03$). Since both Chol and BMI were significantly correlated with elevated liver function parameters (CC 0.5; $P=0.001$) the following analyses were carried out for patients with normal liver functions separately.

Adrenal androgens were correlated with Chol levels and BMI: DHEAS with Chol adjusted for age (CC 0.51; $P=0.005$) and with BMI in patients <40 years (CC 0.6; $P=0.02$). Age-stratified correlation coefficients for DHEA with BMI and and with Chol in patients <40 years were 0.74 ($P=0.009$) and 0.53 ($P=0.008$) respectively.

Sex gonadal hormones correlated with possible cardiovascular risk factors only in patients >30 years of age: E2 with BMI (CC −0.4; NS), E1 with Chol (CC 0.48; $P=0.03$) and with BMI (CC −0.51; NS); T correlated negatively with both BMI and Chol (CC s−0.49 and −0.47 respectively; NS), and 17-OH-pr with Chol (CC −0.47; $P=0.044$).

A trend towards a correlation between treatment modalities and cardiovascular risk factors such as BMI and Chol levels was observed. The cumulative dose of cisplatin and Chol level elevations were significantly correlated only in patients <28 years ($P=0.05$).

### Discussion

In 1948 Spitz was the first to describe testicular atrophy in patients treated with nitrogen mustard for lymphoma. Consecutive studies in patients with lymphoma have particularly investigated the degree of exocrine gonadal damage due to ctx (Bokemeyer *et al.*, 1984). In the last decade long-term toxicity has also become relevant to patients with testicular cancer, as the use of combination ctx and the introduction of cisplatin (Einhorn and Donohue, 1977) have resulted in potentially curative treatment. Yet, data on endocrine toxicity in patients with testicular tumours have mainly focused on fertility issues, and only a few investigations were concerned with additional long-term endocrinological abnormalities of hormone metabolism. Besides sporadically documented minor thyroid-stimulating hormone elevations (Bosl and Bajorunas, 1987; Gietema *et al.*, 1992; Leitner *et al.*, 1986; Schwabe *et al.*, 1992), alterations in oestradiol levels have been found in patients treated for testicular cancer (Gietema *et al.*, 1992; Schwabe *et al.*, 1992), but the impact of these findings, e.g. on lipid profiles and further metabolic changes, were not investigated.

Although patients with testicular cancer may have an increased incidence of pre-existing gonadal disorders (Carroll *et al.*, 1987), it is generally accepted that ctx can significantly alter the gonadal function (Bosl and Bajorunas, 1987). Other factors influencing the regulation of the pituitary–testicular hormone axis may be the malignancy itself, which can adversely affect gonadal function (Blackman *et al.*, 1988) and the secretion of $\beta$-hCG by the tumour, which may influence gonadotropin levels (Cochran *et al.*, 1975). However, these tumour-associated factors can be disregarded in long-term surviving patients after ctx. An influence of unilateral orchidectomy on sex hormone steroid levels has been denied (Hoeppner *et al.*, 1986). Therefore, the present study investigates persisting abnormalities in the male steroid hormone equilibrium following different combination ctx protocols for testicular cancer, in order to identify possible endocrinological damage resulting from ctx. Besides the impact of ctx itself, additional variables such as age, time of follow-up, liver metabolism, serum albumin concentration were taken into consideration and all calculations were performed for the total patient population and for appropriately stratified subgroups.

To evaluate testicular function, serum testosterone (T), and the gonadotropins secreted by the pituitary gland, FSH and LH have to be determined (Horton, 1990). Elevated serum FSH concentrations, probably resulting from deficient secretion of inhibin and sex steroids (Horton, 1990), serve as a reliable marker for tubular dysfunction and infertility, as investigations of spermatogenesis after ctx have shown (Fossa *et al.*, 1985; Kreuser *et al.*, 1989). LH levels rise when testosterone production in the testicular Leydig cells falls.

Testosterone is either maintained at a physiological level – the compensated state – or decreases despite elevated LH levels, resulting in hypogonadism.

Despite the pulsatile excretion mode of LH from the pituitary gland, single LH values taken at a set time of day were highly correlated with FSH levels (CC 0.76; $P<0.001$), indicating the usefulness of single determinations of gonadotropins at a fixed time (Bain *et al.*, 1988) and their value as reliable parameters of gonadal damage.

Controversial data have been reported on the issue of return of fertility with time after ctx (Bissett *et al.*, 1990; Drasga *et al.*, 1983; Hansen *et al.*, 1990; Kreuser *et al.*, 1989). Our results of 63% elevated FSH levels at a median time of 42 months after ctx (1–11 years) and the degree of FSH elevation, which correlates highly significantly with the cumulative dose of cisplatin administered, imply that the damage to spermatogenesis persists in the majority of patients. Only two of ten patients with elevated FSH levels who were also studied before ctx had previously shown elevated FSH values. The occurrence of gonadal toxicity correlated significantly with cisplatin-induced neuro- and ototoxicity in our patients, confirming the impact of cisplatin-based ctx on gonadal impairment (Berger *et al.*, 1995; Bokemeyer *et al.*, 1993).

Furthermore, a correlation between cisplatin dose and LH levels was documented. Although normalisation of elevated LH levels with the time of follow-up has been suggested (Leitner *et al.*, 1986), a relevant decrease in LH and FSH level elevation was seen only in the subset of our patients who had received rather toxic high-dose platinum-based regimens resulting in initially very high gonadotropin levels. In all other treatment groups long-term median LH and FSH elevations persisted or even slightly increased as compared with levels of patients with short observation periods. Thus, our findings do not help enlighten this controversial issue.

A total of 24% of our patients showed endocrinological profiles of a compensated, and 10% of patients of a decompensated, Leydig cell insufficiency. Regarding the median age of 31 years of these patients and the average follow-up of 37 months, impaired Leydig cell function in one-third of all treated patients represents a considerable long-term toxicity. Although the testosterone levels did not decrease any further with the time of follow-up, it is too early to conclude that the compensated Leydig cell insufficiency will return to normal or at least remain stable with time in *all* patients. Since the median testosterone levels were lower after more toxic regimens – in addition to the physiological decrease in testosterone with age – the increased risk of ctx-treated patients for developing premature Leydig cell insufficiency should be kept in mind.

Most data concerning ctx-related gonadal toxicity for testicular cancer are based on PVB ctx (Einhorn and Donohue, 1977). Although a similar degree of gonadal toxicity has been postulated for vinblastine (V) and etoposide (E) used as single agents (Hansen *et al.*, 1990), the current standard regimen for PEB (replacing V by E) was shown to be significantly less gonadotoxic in our patients compared with PVB therapy, when similar cumulative doses of P and B were given. The highest levels of gonadotropins were reached in patients treated with regimens containing both E *and* V or high-dose P.

It has been postulated that male patients over 25 years of age treated with ctx may be more susceptible to persisting gonadal impairment than younger patients (Horwich *et al.*, 1995; Leitner *et al.*, 1986). A toxicity–age correlation could be seen for FSH levels in our patients and statistically significant differences of LH and FSH elevations were found in patients $<28$ *vs* $\geqslant 28$ years after treatment when low-toxicity regimens were considered. As this result is not consistent for all patient groups and as the physiological increase of gonadotropins with age may represent a bias for interpretation (Blackman *et al.*, 1988), this issue will need further investigation.

As corticosteroids cause oligospermia and low sperm motility (Mancini *et al.*, 1966), we analysed gonadal toxicity with respect to the use of high-dose antiemetic steroids during therapy. No influence of steroids could be established. In a study by Hendry *et al.* in 1983 patients with severely depressed sperm counts before ctx had shown a greater potential for recovery after ctx than those with normal counts. However, pharmacological gonadal protection with LH-releasing hormone analogues applied during ctx has so far failed to achieve the protection promised by earlier experimental studies (Krause and Pflueger, 1989). Accordingly, in our patients, suppressed FSH levels, as noted in 3 of 14 patients screened before ctx, did not protect against gonadal toxicity.

17$\alpha$-OH-progesterone constitutes quantitatively the second steroid secreted from the testis (Horton, 1990). Its function in man is not known – it does not seem to be a sex steroid or a regulator of gonadotropins. Therefore, our observation that 17$\alpha$-OH-progesterone levels correlated with LH and testosterone, and increased significantly with the extent of tubular damage remains without clinical interpretation.

DHEAS, a $\Delta$5-androgen, is the major secretory steroidal product of the adrenal gland. Together with its unconjungated form, DHEA, these adrenal androgens had traditionally been considered to be solely prohormones of stronger sex steroids. Recently, it has become clear that, by means of steroid sulphotransferase/sulphate interconvertable hormones, they themselves participate in a striking number of physiological and pathological processes (Parker, 1995), although gross symptoms of clinical withdrawal following experimental adrenalectomy are not observed (Regelson *et al.*, 1994). The metabolism of DHEAS with its unique physiological age-dependent decline after a peak at the age of approx 24 years (Orentreich *et al.*, 1984) has been considered as a marker for life expectancy and biological ageing (Barrett-Connor *et al.*, 1986). Despite a circadian rhythm, single-spot samples measured at a standardised time of day can give representative information on DHEAS levels (Bain *et al.*, 1988).

In our patients DHEAS levels were negatively correlated with age as expected, but DHEA levels failed to show this age-dependent fluctuation. However, DHEA levels correlated highly significantly to the cumulative P dose applied, as well as to LH and FSH levels. Furthermore, an influence of the cumulative doses of E and V was established, postulating an effect of combination ctx on DHEA metabolism, thus altering normal age ranges. Despite the high variability in DHEA level measurements (Zumoff *et al.*, 1980), correlation coefficients from 0.51 to 0.66 with levels of statistical significance from $P<0.001$ to $<0.006$ for DHEA levels and toxicity parameters in our patients indicate more than coincidental hormone distributions.

Since DHEA/DHEAS ratios are not constant interindividual variables, but rather dependent on numerous known – e.g. the extremely interindividual variable activity of the converting enzyme steroid sulphotransferase (Aksoy *et al.*, 1993) – and unknown factors (Liu *et al.*, 1990; Zumoff *et al.*, 1980), the influence and mechanisms of 'treatment toxicity' on DHEA/S levels are difficult to interpret. LeBlanc *et al.* (1992) and Maines *et al.* (1990) have shown that cisplatin has an impact on steroidogenic pathways by influencing the regulation of the cytochrome system as well as altering the testicular mitochondrial sidechain cleavage activity. Yet, the exact sites and definite mechanisms of metabolic dysregulation, especially in long-term toxicity, have not been determined and especially the clinical significance of possible interference with the physiological age-distribution of the adrenal androgens will remain to be investigated.

Oestrogens and androgens may play an important role in the development of risk factors for cardiovascular disease and the association of these steroid hormones with lipid profiles (Haffner *et al.*, 1993), degree of obesity and fat distribution (Pasquali *et al.*, 1991) appears to be established. Oestrogens are associated with increased levels of high-density lipoprotein (HDL) cholesterol, which may decrease the cardiovascular risk (Jacobs *et al*., 1990). Recently, two publications have evaluated the treatment-related cardiovascular risk profile in patients with testicular cancer (Gietema *et al*., 1992; Raghavan *et al*., 1992). Gietema *et al*. demonstrated that cholesterol levels and body fat increased significantly in patients <29 years after ctx compared with patients treated with orchidectomy alone. Elevated cholesterol levels have also been mentioned in studies of long-term toxicity after ctx for testicular cancer (Boyer *et al*., 1990; Schwabe *et al*., 1992). The frequency of cholesterol elevations in 32% of our patients was accordingly higher than would normally be expected in a rather young population and age-stratified comparisons with reference groups (Assmann *et al*., 1986) showed significantly higher cholesterol levels especially in the 19–29 year-old patients. This finding is of concern as an increased incidence of arterial hypertension and of myocardial infarctions after ctx for testicular cancer has been postulated (Berger *et al*., 1995; Bissett *et al*., 1990; Gietema *et al*., 1992; Roth *et al*., 1988; Schwabe *et al*., 1992). Our results further demonstrate the existence of complex relationships between oestrogens, testicular, adrenal steroids, total cholesterol and BMI. This is important as DHEA and the oestrogens E1 and E2 were directly correlated with type and dosage of cytotoxic agents, implying that ctx treatment factors, in addition to patient characteristics such as age and liver function, may have a significant influence on cardiovascular risk profiles.

In conclusion, major endocrinological abnormalities persist in more than half of young patients cured from testicular cancer by cisplatin-based combination ctx regimens. Although the complex interaction between hormones and ctx variables is apparent, it is difficult to establish causal relationships. None of our patients presented with obvious clinical symptoms, which was not surprising as an acquired complete Leydig cell dysfunction would cause physical changes to appear only very slowly in previously sexually mature men, or as a major decline of the adrenal androgens would not produce gross clinical symptoms. Yet, the absence of apparent clinical toxicity does not exclude the possibility of ctx-related secondary morbidity. Subtle shifts in hormonal ratios might not only represent an important factor for increased cardiovascular risk, but might additionally affect other body systems with unknown long-term consequences, while routine screening continuously presents normal serum testosterone levels. In order to recognise endocrinological alterations after ctx, extensive investigation may be necessary. With the increasing use of high-dose ctx, made possible by overcoming haematological toxicity through stem cell rescue, long-term alterations of the hormonal equilibrium may become even more important. In 1972 Campos postulated that generalised treatment causes what he called 'continuous positive ageing' – delaying tumour death through treatment, but making a 'physiological' death more likely with the passage of time. Surveillance and adequate supportive measures should be able to assure that the years of life gained by therapy are not lost to toxicity.

**Acknowledgements**
We thank H Geerlings from the Institute of Biometry, Hannover University Medical School for his advice in statistical analysis.

### References

AASS N, KAASA S, LUND E, KAALHUS O, SKARD HEITER M AND FOSSA SD. (1990). Long-term somatic side-effects and morbility in testicular cancer patients. *Br. J. Cancer*, **61**, 151–155.

AKSOY IA, SOCHOROVA V AND WEINSHILBOUM RM. (1993). Human liver dehydroepiandosterone sulphotransferase: nature and extent of individual variation. *Clin. Pharmacol. Ther.*, **54**, 498–506.

ASSMANN G AND SCHULTE H. (1986). *PROCAM Studie*. Panscientia: Zurich.

BAIN JR, LANGERIN M, D'COSTA R, SANDS M AND HUCHER S. (1988). Serum-pituitary and steroid hormone levels in the adult male: one value is as good as three. *Fertil. Steril.*, **49**, 123–126.

BARRETT-CONNOR E AND KHAW K-T. (1988). Endogenous sex hormones and cardiovascular disease in men. *Circulation*, **78**, 539–545.

BARRETT-CONNOR E, KHAW K-T AND YEN SSC. (1986). A prospective study of DHEAS, mortality, and cardiovascular disease. *N. Engl. J. Med.*, **315**, 1519–1524.

BERGER CC, BOKEMEYER C, SCHNEIDER M, KUCZYK MA AND SCHMOLL H-J. (1995). Secondary Raynaud's phenomenon and other late vascular complications following chemotherapy for testicular cancer. *Eur. J. Cancer*, **31 A**, 2229–2238.

BISSETT D, KUNKELER L, ZWANENBURG L, PAUL J, GRAY C, SWAN IRC, KERR DJ AND KAYE SB. (1990). Long-term sequelae of treatment for testicular germ cell tumour. *Br. J. Cancer*, **62**, 655–659.

BLACKMAN MR, WEINTRAUB BD, ROSEN SW AND HARMANN SM. (1988). Comparison of the effects of lung cancer, benign lung disease, and normal aging on pituitary–gonadal function in men. *J. Clin. Endocrinol. Metab.*, **66**, 88–95.

BOKEMEYER C, BERGER CC, KYNAST B, SCHMOLLL H-J AND POLIWODA H. (1993). Ototoxicity following therapy for testicular cancer. *Eur. J. Cancer*, **29A**, (suppl. 6). 1357.

BOKEMEYER C, SCHMOLL H-J, VON RHEE J, KUCZYK M, SCHUPPERT F AND POLIWODA H. (1994). Long-term gonadal toxicity after therapy for Hodgkin's and non-Hodgkin's lymphoma. *Ann Hematol.*, **68**, 105–110.

BOSL GJ AND BAJORUNAS D. (1987). Pituitary and testicular hormonal function after treatment for germ cell tumour. *Int. J. Androl.*, **10**, 381–384.

BOYER M, RAGHAVAN D, HARRIS PJ, LIETCH J, BLEASEL A, WALSH JC, ANDERSON S AND TSANG C-S. (1990). Lack of late toxicity in patients treated with cisplatin-containing combination chemotherapy for metastatic testicular cancer. *J. Clin. Oncol.*, **8**, 21–26.

CAMPOS JL. (1972). Continuous positive aging in Hodgkin's disease. *Br. J. Radiol.*, **45**, 917–922.

CARROLL PR, WHITMORE JR W, HERR H, MORSE M, SOGANI P, BAJORUNAS D, FAIR WR AND CHAGANTI RSK. (1987). Endocrine and exocrine profiles of men with testicular tumours before orchidectomy. *J. Urol.*, **137**, 420–423.

COCHRAN JS, WALSH PC, PORTER JC, NICHOLSON TC, MADDEN JD AND PETERS DC. (1975). The endocrinology of human chorion gonadotropin-secreting testicular tumours: a new method in diagnosis. *J. Urol.*, **114**, 549.

DRASGA RE, EINHORN LH, WILLIAMS SD, PATEL DN AND STEVENS EE. (1983). Fertility after chemotherapy for testicular cancer. *J. Clin. Oncol.*, **1**, 179–183.

EINHORN LA AND DONOHUE J. (1977). Cis-diamminedichloroplatinum, vinblastine, and bleomycin combination chemotherapy in disseminated testicular cancer. *Ann. Intern. Med.*, **87**, 293–298.

ELLIS PA, GEORGE PM, ROBINSON BA, ATKINSON CH AND COLLS BM. (1992). Fasting plasma lipid measurements following cisplatin chemotherapy in patients with germ cell tumours. *J. Clin. Oncol.*, **10**, 1609–1614.

FOSSA SD, OUS S, ABYHOLM T, NORMAN N AND LOEB M. (1985). Post-treatment fertility in patients with testicular cancer. (II). *Br. J. Urol.*, **57**, 210–214.

GIETEMA JA, SLEIJFER DTH, WILLEMSE PHB AND SCHRAFFORDT KOOPS H. (1992). Long-term follow-up of cardiovascular risk factors in patients given chemotherapy for disseminated nonseminomatous testicular cancer. *Ann. Intern. Med.*, **116**, 709–715.

GIONA F, ANNINO L, DONATO P AND ERMINI M. (1994). Gonadal, adrenal, androgen and thyroid functions in adults treated for acute lymphoblastic leukemia. *Haematologica.*, **79**, 141–147.

HAFFNER SM, MYKKANEN L, VALDEZ RA AND KATZ MS. (1993). Relationship of sex hormones to lipids and lipoproteins in nondiabetic men. *J. Clin. Endocrinol. Metab.*, **77**, 1610–1615.

HANSEN SW, BERTHELSEN JG AND VON DER MAASE H. (1990). Long-term fertility and leydig cell function in patients treated for germ cell cancer with cisplatin, vinblastine, and bleomycin versus surveillance. *J. Clin. Oncol.*, **8**, 1695–1698.

HENDRY WF, STEDRONSKA J, JONES CR, BLACKMORE CA, BARRETT A AND PECKHAM MJ. (1983). Semen analysis in testicular cancer and Hodgkin's disease: pre- and post-treatment findings and implications for cryopreservation. *Br. J. Urol.*, **55**, 769–773.

HOEPPNER W, REINEL D AND HARTMANN M. (1986). Untersuchungen zur Fertilitaet von Patienten mit malignen Hodentumoren zum Zeitpunkt der Orchidektomie. *Andrologica*, **18**, 398–405.

HOLMES SJ, WHITEHOUSE RW, CLARK ST, CROWTHER DC, ADAMS JE AND SHALET SM. (1994). Reduced bone mineral density in men following chemotherapy for Hodgkin's disease. *Br. J. Cancer*, **70**, 371–375.

HORTON RJ. (1990). Testicular steroid transport, metabolism and effects. In *Principles and Practice of Endocrinology and Metabolism*. Becker KL (ed.) pp.937–941. Lippincott: Philadelphia.

HORWICH A, LAMPE H, NORMAN A, NICHOLLS J, JAY G AND DEARNALEY D. (1995). Fertility after chemotherapy for metastatic germ cell tumours. *Proc. ASCO.*, **14**, 235.

JACOBS DR, MEBANE IL, BANGDIWALA SI, CRIQUI MH AND TYROLER HA. (1990). High density lipoprotein cholesterol as a predictor of cardiovascular disease mortality in men and women: the follow-up study of the Lipid Research Clinics Prevalence Study. *Am. J. Epidemiol.*, **131**, 32–47.

KRAUSE W AND PFLUEGER KH. (1989). Treatment with gonadotropin-releasing-hormone agonist buserelin to protect spermatogenesis against cytotoxic treatment in young men. *Andrologia*, **21**, 265–270.

KREUSER ED, KURRLE E, HETZEL WD, HETMER B, PORZSOLT F, HAUTMANN R, GAUS W, SCHLIPF U, PFEIFFER EF AND HEIMPEL H. (1989). Reversible Keimzelltoxizität nach aggressiver Chemotherapie bei Patienten mit Hodentumoren: Ergebnisse einer prospektiven Studie. *Klin. Wochenschr.*, **67**, 367–378.

LEBLANC GA, KANTOFF PW, FREI E AND WAXMAN DJ. (1992). Hormonal perturbations in patients with testicular cancer treated with cisplatin. *Cancer*, **69**, 2306–2310.

LEITNER SP, BOSL GJ AND BAJORUNAS D. (1986). Gonadal dysfunction in patients treated for metastatic germ cell tumour. *J. Clin. Oncol.*, **4**, 1500–1505.

LIU CH, LAUGHLIN GA, FISCHER UG AND YEN SS. (1990). Marked attenuation of ultradian and circadian rhthyms of dehydroepiandrosterone in postmenopausal women: evidence for a reduced 17, 20-desmolase enzymatic activity. *J. Clin. Endocrinol. Metab.*, **71**, 900–906.

MAINES MD, SLUSS PM AND ISCAN M. (1990). *cis*-Platinum-mediated decrease in serum testosterone is associated with depression of luteinizing hormone receptors and cytochrome P-$450_{scc}$ in rat testis. *Endocrinology*, **126**, 2398–2406.

MANCINI RE, LAVIERI JC, MULLER F, ANDRADA HA AND SARACENI DJ. (1966). Effect of prednisolone upon normal and pathologic human spermatogenesis. *Fertil. Steril.*, **17**, 500.

NEIL HAW, MANT D, JONES L, MORGAN B AND MANN JI. (1990). Lipid screening: is it enough to measure total cholesterol concentration? *Br. Med. J.*, **301**, 584–587.

ORENTREICH N, BRIND JL, RIZER RL AND VOGELMAN JH. (1984). Age changes and sex differences in serum dehydroepiandrosterone sulfate concentrations throughout adulthood. *J. Clin. Endocrinol. Metab.*, **59**, 551–555.

PARKER LN. (1995). Adrenal Androgens. In *Endocrinology*, De Groot LJ. (ed.) pp. 1836–1851. W.B. Saunders: Philadelphia.

PASQUALI R, CASIMIRRI F, CANTOBELLI S, MELCHIONDA N, LABATE AMM, FABBRI R, CAPELLI M AND BORTOLUZZI L. (1991). Effect of obesity and body fat distribution on sex hormones and insulin in men. *Metabolism*, **40**, 101–104.

RAGHAVAN D, COX K, CHILDS A, GRYGIEL J AND SULLIVAN D. (1992). Hypercholesterolemia after chemotherapy for testis cancer. *J. Clin. Oncol.*, **10**, 1386–1389.

REGELSON W, LORIA R AND KALIMI M. (1994). Dehydroepiandrosterone (DHEA) - the 'mother steroid'. *Ann. N.Y. Acad. Sci.*, **719**, 553–563.

ROTH BJ, GRIEST A, KUBILIS PS, WILLIAMS SD AND EINHORN LH. (1988). Cisplatin-based combination chemotherapy for disseminated gem cell tumour: long-term follow-up. *J. Clin. Oncol.*, **6**, 1239–1247.

SCHWABE H-R, HERRMANN R, MATHEW M, GRAEF K-J, SANDER T, CORDES M, NAGEL R, WEISSBACH L AND HUHN D. (1992). Langfristige Toxizitaet der Polychemotherapie bei kurativ behandeltem Hodenkarzinom. *Dtsch. Med. Wschr.*, **117**, 121–126.

SPITZ S. (1948). The histological effects of nitrogen mustards on human tumours and tissues. *Cancer*, **1**, 383.

ZUMOFF B, ROSENFELD RS, STRAIN GW, LEVIN J AND FUKUSHIMA DK. (1980). Sex differences in the 24-hour mean plasma concentrations of dehydroisoandrosterone (DHA) and dehydroisoandrosterone sulfate (DHS) and the DHA to DHS ratio in normal adults. *J. Clin. Endocrinol. Metab.*, **51**, 330–333.